# Low-intensity pulsed ultrasound for treating delayed union scaphoid fractures: case series

**DOI:** 10.1186/s13018-015-0221-9

**Published:** 2015-05-20

**Authors:** Uri Farkash, Orit Bain, Arnon Gam, Meir Nyska, Paul Sagiv

**Affiliations:** Central Orthopedic Clinic, IDF, Zerifin, Israel; Department of Orthopedic Surgery, Meir Medical Center, 59 Tchernihovsky St, Kfar-Saba, 44281 Israel

**Keywords:** Ultrasound, Low-intensity pulsed ultrasound (LIPUS), Delayed union, Scaphoid fracture, Bone healing

## Abstract

**Background:**

The standard treatment to enhance fracture healing of scaphoid nonunions is surgery. Low-intensity pulsed ultrasound (LIPUS) is gaining in popularity as an alternative treatment to improve fracture healing; however, little is known about success rates of this treatment in scaphoid-delayed unions. The purpose of our study is to define the success rate of LIPUS treatment for delayed union of scaphoid fractures and further analyze whether initial management or fracture type influences success rate.

**Methods:**

During the period of 2011–2013, in the central orthopedic clinic of our institution, patients diagnosed with delayed union of the scaphoid were offered with LIPUS treatment as an alternative to conventional surgical treatment. These patients were then divided into subgroups according to the time elapsed from initial injury until diagnosis of the fracture.

**Results:**

Overall, 22 of 29 (76 %) fractures healed, 12 of 13 (92 %) of the early diagnosed group, and 10 of 16 (63 %) of the late diagnosed group. Difference in healing rate between proximal pole, waist, and distal pole fractures was not statistically significant.

**Conclusion:**

LIPUS can help heal delayed union scaphoid fractures, especially in fractures diagnosed and treated soon after injury and may serve as an alternative to surgical treatment.

## Background

The scaphoid is the most commonly fractured carpal bone, accounting for 60 to 70 % of all carpal fractures, and second in frequency only to distal radius fractures [1]. The majority of injuries are low-energy injuries [2, 3], and tend to occur in young adult men between the ages of 15 and 40 years. The importance of a correct diagnosis and appropriate treatment of these fractures lie in the scaphoid’s tenuous blood supply, which may explain the increased frequency of delayed union, nonunion, and avascular necrosis (AVN) of scaphoid fractures. If left untreated, scaphoid nonunions can progress to a predictable pattern of radiocarpal arthrosis [4].

The goal of treatment should be a consolidation of the fracture with the scaphoid in anatomic alignment. Because of evidence linking nonunions with osteoarthritis, surgery is recommended for most young, healthy patients, even if they are free of symptoms and have normal wrist mobility. Most hand surgeons recommend open reduction and internal fixation of the nonunion combined with a bone graft [5, 6]. This treatment results in a union rate as high as 97 % [7].

In order to reduce complications of fracture care, there is great incentive to develop therapies that will accelerate bone healing. Our team performed a preliminary study which yielded a 91 % healing rate for scaphoid delayed unions treated with low-intensity pulsed ultrasound (LIPUS) initiated up to 1 year after the fracture and a drop in union rate to 29 % if treatment began more than 1 year after the fracture occurred (unpublished observations, 2009). This preliminary study led to a treatment protocol in our institution, offering LIPUS treatment for patients suffering from scaphoid delayed unions, up to 1 year after injury.

The purpose of our study was to define the success rate of LIPUS treatment for delayed union of scaphoid fractures and to further analyze whether initial management or fracture type influences success rate.

## Patients and methods

Institutional review board approval was not required for this study. During the period of 2011–2013, patients in the central orthopedic clinic of our institution were offered with LIPUS treatment as an alternative to conventional surgical treatment when delayed union of the scaphoid was diagnosed. Delayed union was defined if 3 months to 1 year elapsed from the initial injury with no signs of fracture healing (no trabeculae crossing the fracture line) or if the fracture was only tenuously united (bridging trabeculae in less than 50 % of the cross section, according to the method described by Singh et al. [8], based on CT scan). Patients who elected LIPUS instead of surgical treatment were included in the study. Fractures displaced >1 mm or having humpback deformity were excluded, and operative treatment was recommended.

Data regarding patient demographics, date and mechanism of injury, date of fracture diagnosis, type of fracture, and initial treatment were collected from patients’ records.

Patients were divided into subgroups according to the time elapsed from initial injury until fracture diagnosis. Early diagnosed group included patients with fractures diagnosed soon after injury (usually within a few days and less than 3 months from injury). This group of patients was treated by immobilization in a thumb spica cast. LIPUS treatment was added 3 months after the injury. Late diagnosed group included patients with fractures that were diagnosed more than 3 months after the injury. In this group, thumb spica cast was installed and LIPUS treatment was initiated at the time of diagnosis.

**Fig. 1:**
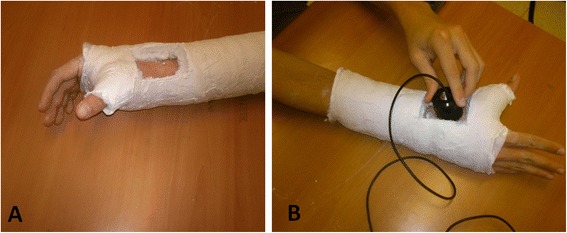
LIPUS treatment. **a** Thumb spica cast with a “window”. **b** Application of LIPUS device through the “window” in the cast

All patients were treated by ultrasonic therapy device (Melmak GmbH, Raisting, Germany), with a daily 20-min dose of pulsed, low-intensity (SATA) 30 mW/cm^2^ ultrasound, frequency of 1.5 MHz, and signal pulse duration of 200 μs, repetition rate of 1.0 KHz. The treatment was administered through a window in the cast (Fig. 1).

Follow-up examination was undertaken using repeated X-rays or CT scans until fracture healing was achieved (bridging trabeculae of at least 75 % of the cross section, according to the method described by Singh et al. [8]), or until no signs of improvement in fracture healing were recorded, LIPUS was terminated, and surgical treatment was advised.

Patient characteristics for comparing early versus late diagnosed groups were described by percentages and means. Continuous variables were compared using independent Student’s *t* test, and categorical variables were compared using chi-square test or Fisher’s exact test. A *P* value < 0.05 was considered statistically significant.

## Results

A total of 29 male patients were included in the study, all aged 18–22, except for one aged 34. Sixteen fractures were in the right hand. Most of the injuries (22) were from a fall on an outstretched hand, usually during sports activity. Other fractures resulted from direct trauma to the wrist. Seventeen fractures were in the scaphoid waist, eleven in the proximal third of the bone, and one in the distal third.

Thirteen patients were diagnosed early (average 5.5 days after injury, range 0–17 days). They were treated with thumb cast immobilization at diagnosis, and 3 months after injury showed no signs of healing (six patients) or inadequate partial healing (less than 50 % of bridging at the fracture site) considered insufficient to discontinue cast treatment (seven patients), before LIPUS was initiated.

Late diagnosed group included 16 patients. Their fracture was diagnosed at an average of 7 months (range 3–12) after injury, and they were treated by cast and LIPUS at the time of diagnosis. Although this group received no initial treatment after the injury, two patients in this group showed signs of partial (less than 50 %) healing at diagnosis.

Average treatment duration of LIPUS was 2.3 months (range 1–4). The average was 2.0 for the early diagnosed group and 2.6 for the late diagnosed group. Average treatment duration of the healed patients was 2.2 months and for the non-healed patients, 2.6 months.

**Fig. 2:**
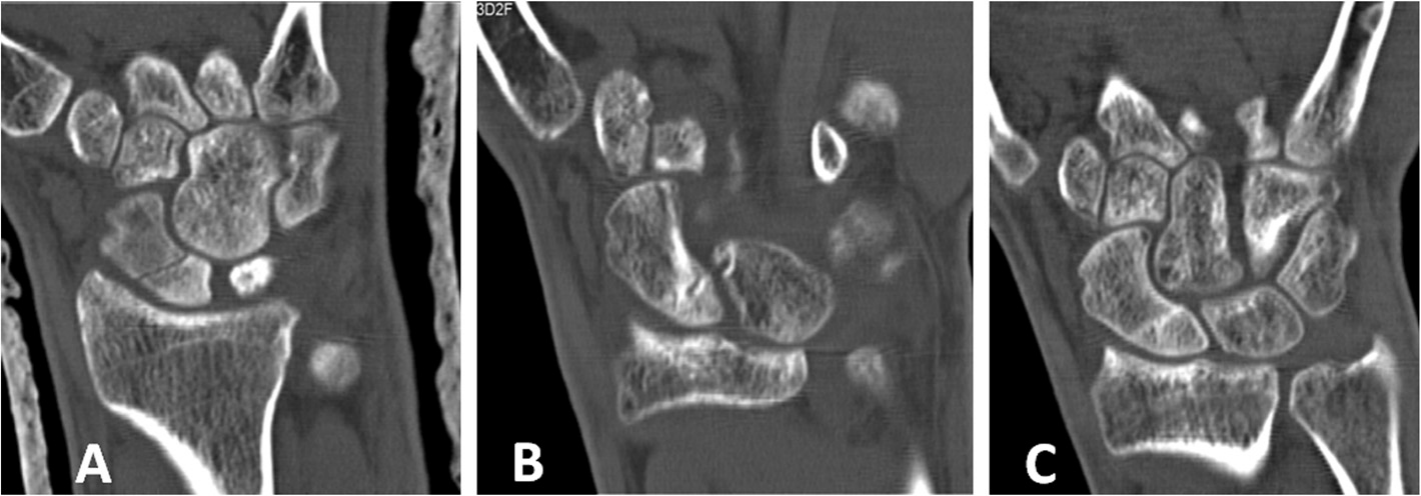
Early diagnosed group. **a** Fracture of the waist of the scaphoid diagnosed shortly after the injury and treated with thumb spica cast. Three months after injury, CT scan shows no signs of fracture healing. LIPUS treatment initiated. **b** After 2 months of LIPUS treatment, partial healing of the fracture. **c** After additional month of LIPUS treatment, complete fracture healing

**Fig. 3:**
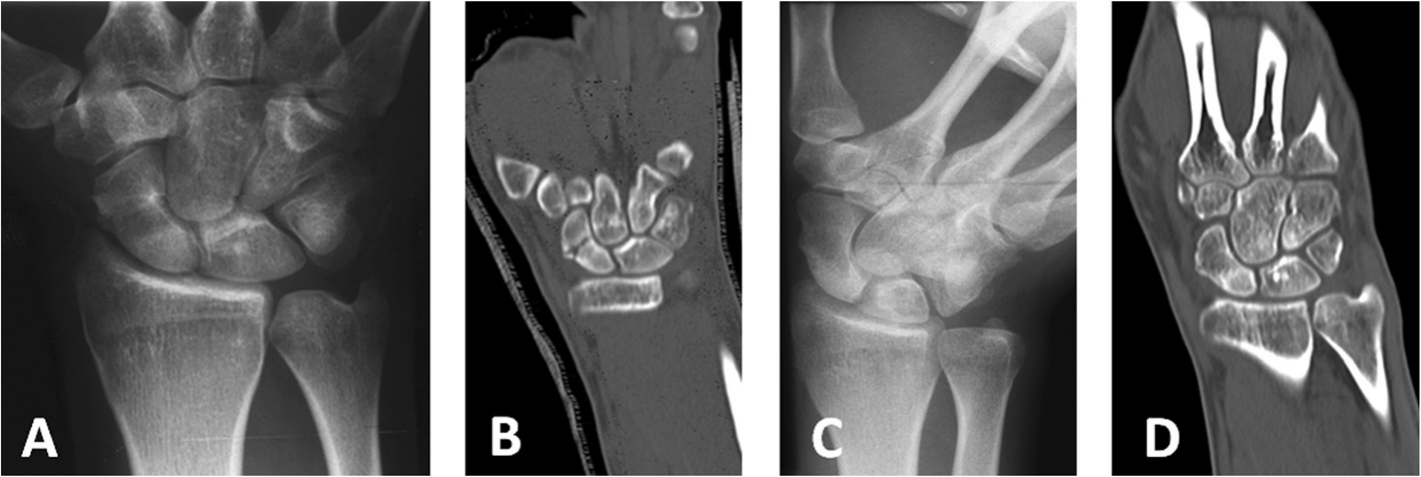
Early diagnosed group. **a** Fracture of the waist of the scaphoid, incurred during contact sport, diagnosed 2 weeks after initial injury. **b** CT scan after immobilization in a thumb spica cast for 3 months, no sign of healing. **c**, **d** X-ray and CT scan of patient’s wrist after 2 months of LIPUS treatment, showing complete fracture healing

**Fig. 4:**
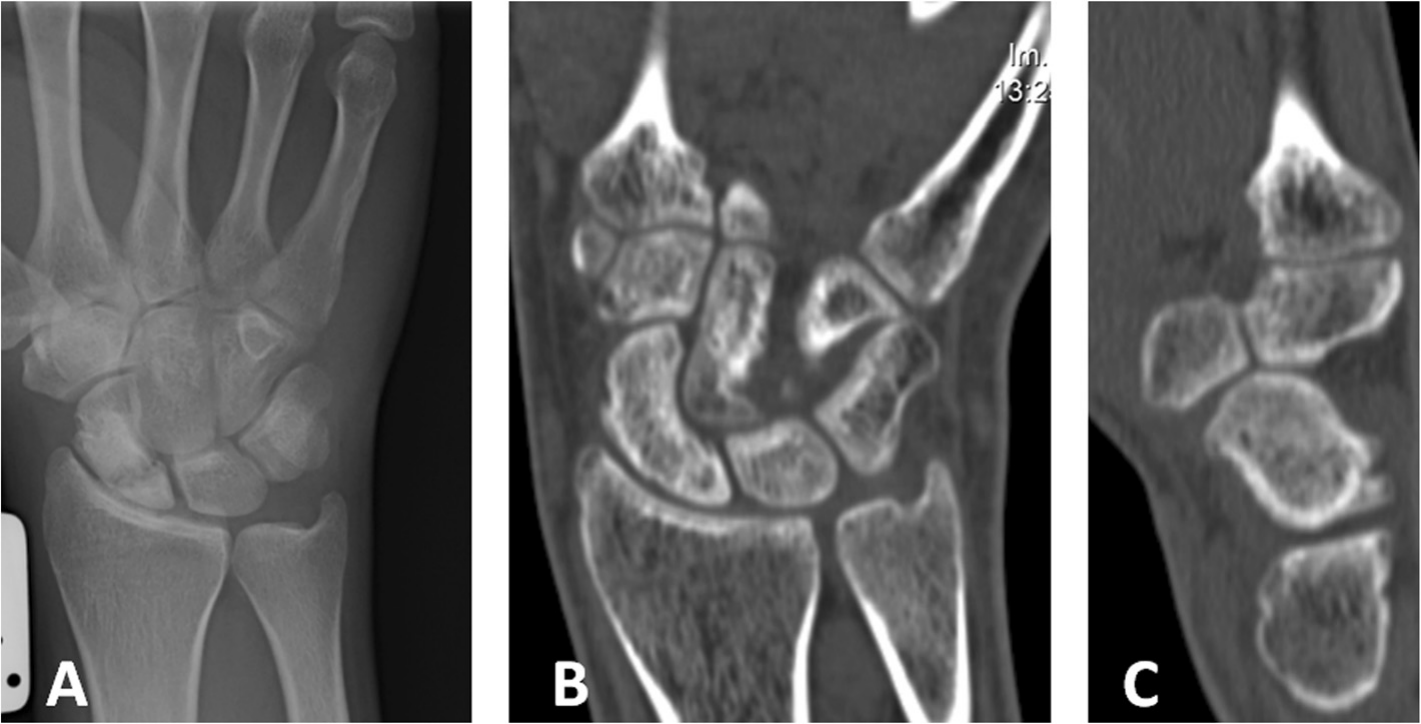
Early diagnosed group. **a** Patient fell and injured his wrist; a fracture of the waist of the scaphoid was diagnosed and cast installed. Three months after cast immobilization, X-ray showed bone resorption and no signs of union of the fracture. LIPUS treatment initiated. **b**, **c** After 3 months of LIPUS treatment, CT scan demonstrates complete healing of the fracture

**Fig. 5:**
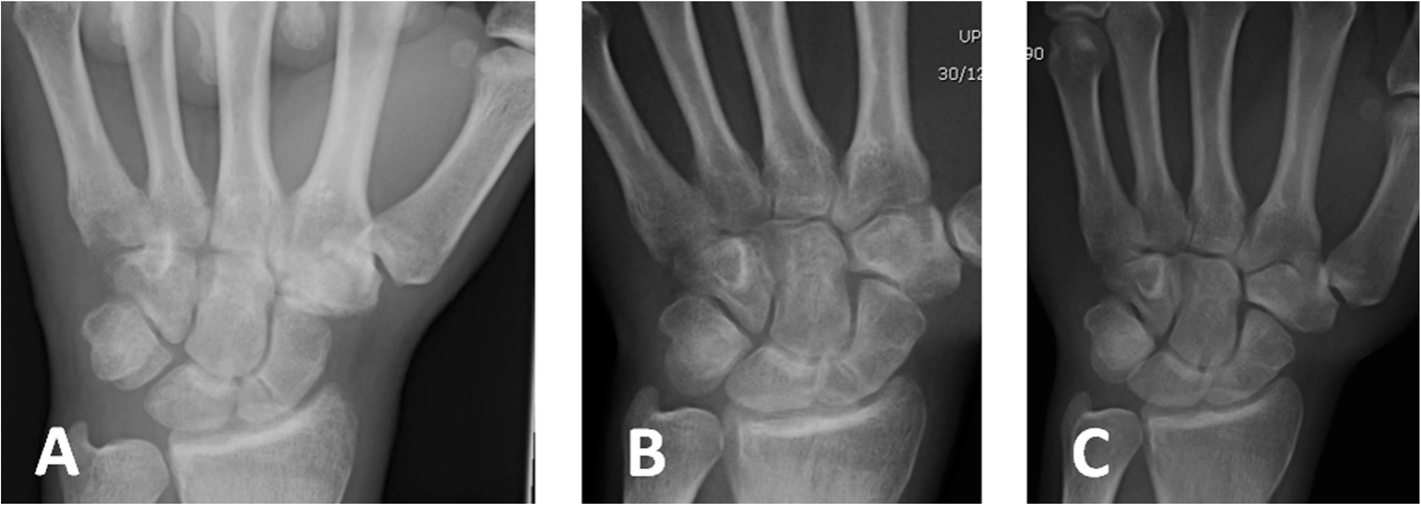
Early diagnosed group. **a** X-ray shows a fracture of the waist of the scaphoid incurred after a fall while playing soccer 2 weeks prior to diagnosis. Thumb spica cast installed. **b** Three months after cast immobilization, X-ray reveals only partial bridging across the fracture line. LIPUS treatment initiated. **c** After a month of LIPUS treatment, complete healing of the fracture

**Fig. 6:**
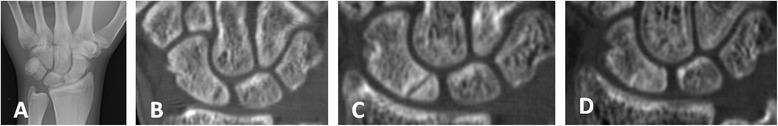
Late diagnosed group. **a** X-ray of patient complaining of wrist pain 3 months after a fall on an outstretched hand showed bone resorption in the proximal pole of the scaphoid. **b** CT scan confirmed the diagnosis of horizontal fracture of the proximal pole of the scaphoid; thumb spica was installed and LIPUS treatment initiated. **c** Six weeks after LIPUS treatment, no signs of bridging across the fracture line. **d** Complete fracture healing after additional month of LIPUS treatment

**Fig. 7:**
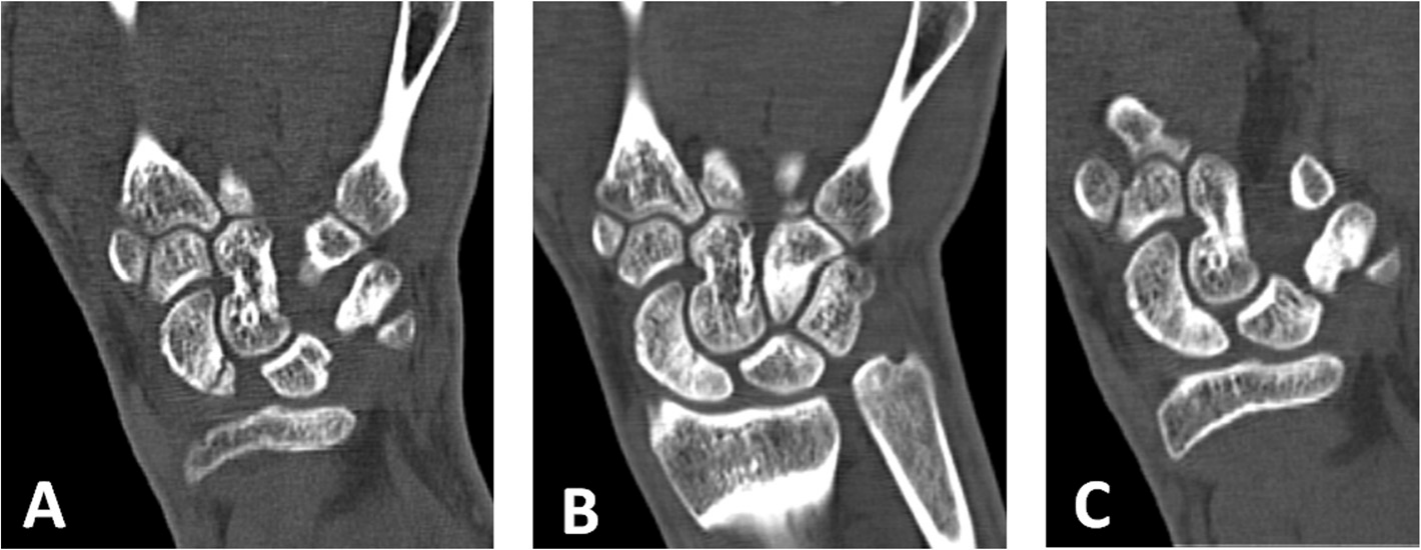
Late diagnosed group. **a** CT scan of the wrist showing a vertical fracture of the proximal pole of the scaphoid, incurred while playing soccer, and diagnosed 3 months after the injury. Cast installed and LIPUS treatment initiated shortly after fracture diagnosis. **b** Two months after LIPUS treatment, incomplete fracture healing. **c** Complete fracture healing after additional month of LIPUS treatment

**Fig. 8:**
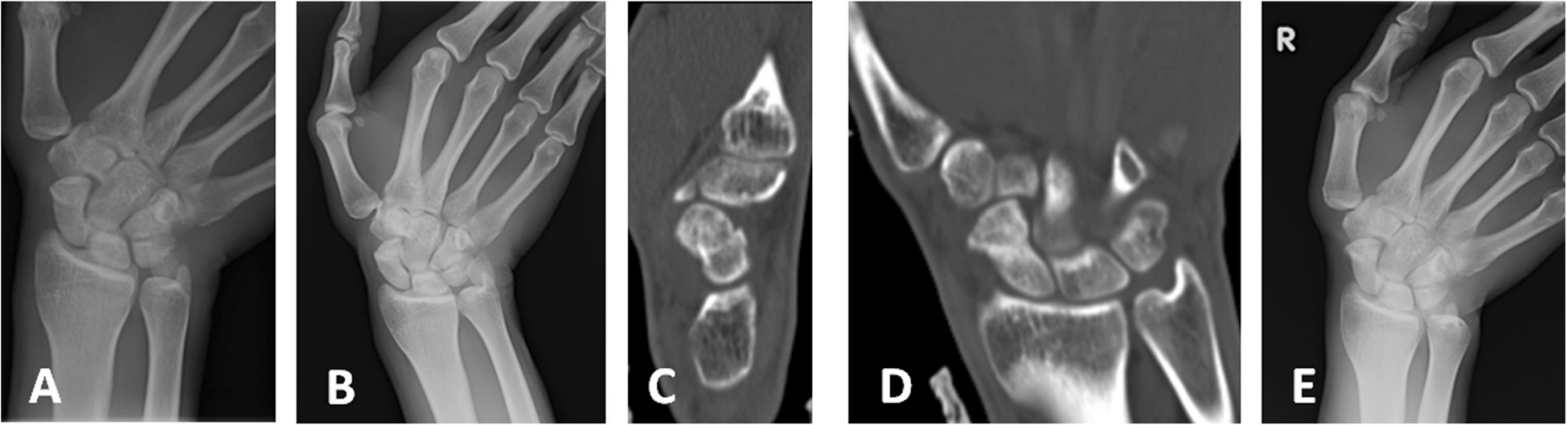
Late diagnosed group. **a** X-ray of the right wrist after injury sustained during martial arts training. No signs of fracture are evident; therefore, patient received no treatment. **b** Seven months after the injury, repeat X-ray revealed fracture of the distal third of the scaphoid. Thumb spica cast was installed and LIPUS treatment began. **c**, **d**, **e** After 2 months of treatment, X-ray and CT scan demonstrate minimally displaced completely healed fracture

Overall, 22 of 29 (76 %) fractures healed, 12 of 13 (92 %) of the early diagnosed group, and 10 of 16 (63 %) of the late diagnosed group (*P* = 0.06). According to fracture location, 9 of 11 (82 %) proximal pole, 12 of 17 (71 %) of waist, and 1 of 1 (100 %) distal pole fractures healed. This difference was not statistically significant. Figs. 2, 3, 4, 5, 6, 7, and 8 show serial X-rays and CT scans of some of the fractures which healed following the LIPUS treatment.

## Discussion

The first report describing the stimulation of bone formation using ultrasound waves was published by Maintz [9] in 1950. This report showed that, although ultrasound at high intensities caused thermal damage, lower doses led to new periosteal bone formation after radius osteotomy in rabbits. LIPUS has minimal thermal effects due to its low intensity [10]. Xavier and Duarte [11] were the first to report the successful use of LIPUS for human fractures. Subsequently, several reports demonstrated the positive influence of LIPUS on fracture healing, whereas other studies did not support its effectiveness. Bashardoust Tajali [12] published a systematic review and meta-analysis regarding the effects of LIPUS therapy on fracture healing. They reviewed 23 studies that used LIPUS stimulation on a variety of bone injuries. Interestingly, similar ultrasound parameters consisting of 20 min of daily LIPUS signal were used by all investigators. They identified that LIPUS therapy stimulates radiographic bone healing in fresh fractures but for delayed unions and nonunion evidence in favor of LIPUS effectiveness was weak [12].

Our knowledge of the effect of LIPUS on scaphoid fracture healing is limited. In 2000, Mayr et al. performed a randomized controlled trial of 30 patients, investigating the role of LIPUS in the treatment of acute scaphoid fractures [13]. Fifteen patients treated with cast immobilization and LIPUS were compared to 15 patients treated with casting alone until radiographic consolidation. In that randomized study, there was a statistically significant decrease (30 %) in time to radiographic healing in the LIPUS treatment group.

The most often cited study of LIPUS treatment for delayed union scaphoid fractures is a retrospective review of 933 delayed unions and 366 nonunion of various bones, many of which were in the upper extremity by Mayr et al. [14], published in 2000. For delayed unions (mean fracture age, 150 d; range not reported), they reported a healing rate of 95 % in the clavicle, 76 % in the humerus, 94 % in the radius, 81 % in the ulna, and 94 % in the scaphoid. For nonunion (average fracture age, 755 d; range not reported), they reported a healing rate of 80 % in the clavicle, 69 % in the humerus, 95 % in the radius, and 100 % in the scaphoid. The average time to healing was 152 days. The criteria by which union was assessed were not described in their methods. Their study, however, included a mixture of patients; some of them also had surgical treatment. The study does not mention if scaphoid fractures were treated surgically as well. Our study showed a healing rate of 76 % for scaphoid delayed unions. Although this rate is not as high as that reported by Mayr, the results are from a more homogenous group of young patients with a similar diagnosis.

The time from injury until medical assistance and the quality of treatment provided at initial presentation may contribute to scaphoid healing potential [15]. Langhoff and Andersen [16] found that the nonunion rate was 40 % when diagnosis and treatment were delayed by 4 weeks, compared to 3 % when diagnosis and treatment occurred within 4 weeks. Our study shows a trend toward better healing rates in the early diagnosed group of patients. Study of a larger group of patients could probably show a significant difference. These patients were treated initially in a thumb spica cast, and LIPUS was added later in the course of treatment, if union of the fracture did not occur within 3 months. Mayr [17] also noticed better healing rates and shorter treatment duration in his group of patients with scaphoid delayed and nonunions treated with LIPUS, achieving 80 % healing in ten patients treated with cast compared to 67 % healing in six patients without cast.

The limitations of our study were:

- There is a lack of strict criteria for follow-up imaging and treatment failure. Since this is a retrospective study involving several treating physicians, we lack uniformity in the follow-up protocol. However, average treatment duration of the non-healed group was longer than for healed group, suggesting that the inability to achieve fracture healing is probably not related to duration of treatment. There is a possibility that treating for a longer period of time may produce better results. Mayr [14] applied LIPUS therapy on delayed and nonunions in different bones, resulting in healing after a mean of 129 days in the delayed unions and 152 days in the nonunions. Rutten et al. [18] applied LIPUS on post-traumatic tibial nonunion, resulting in healing after a mean of 184 days. Since there are no reports in the literature regarding the results of scaphoid healing after extended periods of immobilization and as our patients had the option of surgical treatment with a known high healing rate, we felt that if the fracture did not heal with 3–4 months of LIPUS treatment, LIPUS should be terminated and surgical treatment should be recommended.
- Evaluation of the efficacy of LIPUS treatment without comparing it to a control group in a randomized control trial is problematic. However, since spontaneous healing was not expected, LIPUS treatment was a real alternative to surgical treatment for all our patients. Each patient can be considered as their own control, and every healed fracture avoided surgical intervention. Although surgery for scaphoid nonunions has a high success rate with favorable long-term results, especially in younger patients where delay between injury and surgery is moderate [19], complications of surgery may be more common than is generally reported [20]; therefore, some patients prefer to avoid surgery and seek conservative treatment options.
- Immobilization of the fracture in a thumb spica cast was an essential part of the treatment. Although many physicians treating scaphoid-delayed unions would abandon cast treatment after 3 months and offer surgical treatment, the efficacy of prolonged immobilization in a cast was never investigated to the best of our knowledge. Therefore, it is impossible to know if the healing effect we observed was due to LIPUS, cast immobilization, or both.

## Conclusion

Our results suggest that LIPUS can help heal delayed union scaphoid fractures, especially in fractures diagnosed and treated soon after injury. LIPUS might serve as an alternative treatment for delayed union scaphoid fractures, for patients who wish to avoid surgical treatment, but careful follow up is needed to ensure healing.

## Abbreviations

LIPUS: Low-intensity pulsed ultrasound
AVN: Avascular necrosis
